# Nonsteroidal Anti-Inflammatory Drugs as PPARγ Agonists Can Induce PRODH/POX-Dependent Apoptosis in Breast Cancer Cells: New Alternative Pathway in NSAID-Induced Apoptosis

**DOI:** 10.3390/ijms23031510

**Published:** 2022-01-28

**Authors:** Adam Kazberuk, Magda Chalecka, Jerzy Palka, Arkadiusz Surazynski

**Affiliations:** Department of Medicinal Chemistry, Medical University of Bialystok, 15-222 Bialystok, Poland; kadam568@gmail.com (A.K.); magda.chalecka@umb.edu.pl (M.C.); jerzy.pal@umb.edu.pl (J.P.)

**Keywords:** proline, proline oxidase, proline dehydrogenase, NSAIDS, PPAR, COX, apoptosis, breast cancer, oxidative stress

## Abstract

Nonsteroidal anti-inflammatory drugs (NSAIDs) are considered to be therapeutics in cancer prevention because of their inhibitory effect on cyclooxygenases (COX), which are frequently overexpressed in many types of cancer. However, it was also demonstrated that NSAIDs provoked a proapoptotic effect in COX knocked-out cancer cells. Here, we suggest that this group of drugs may provoke antineoplastic activity through the activation of PPARγ, which induces proline dehydrogenase/proline oxidase (PRODH/POX)-dependent apoptosis. PRODH/POX is a mitochondrial enzyme that catalyzes proline degradation, during which ATP or reactive oxygen species (ROS) are generated. We have found that NSAIDs induced PRODH/POX and PPARγ expressions (as demonstrated by Western Blot or immunofluorescence analysis) and cytotoxicity (as demonstrated by MTT, cytometric assay, and DNA biosynthesis assay) in breast cancer MCF7 cells. Simultaneously, the NSAIDs inhibited collagen biosynthesis, supporting proline for PRODH/POX-induced ROS-dependent apoptosis (as demonstrated by an increase in the expression of apoptosis markers). The data suggest that targeting proline metabolism and the PRODH/POX–PPARγ axis can be considered a novel approach for breast cancer treatment.

## 1. Introduction

Pharmacoepidemiologic data confirm that patients regularly taking nonsteroidal anti-inflammatory drugs (NSAIDs) decreased their risks for different types of cancers, such as breast, prostate, lung, and colorectal cancers [1,2,3]. Although the complex regulatory mechanisms involved in cancer growth and metabolism are well recognized, the treatment of cancer is still a challenge in oncology. 

Some approaches have focused on the anti-inflammatory treatment of cancer [4]. In fact, the inflammatory environment is favorable for cancer development. Particularly important in this process are cyclooxygenases (COXs), which are the specific enzymes responsible for prostaglandin synthesis from arachidonic and linoleic acids. Two isoforms of this enzyme were discovered: COX1, constitutively expressed in most of the cells, and COX2, expressed in response to inflammation. In most cancers, COX2 is overexpressed [5]. It contributes to an increase in the synthesis of prostaglandin E_2_ (PGE_2_), which is involved in cancer cell proliferation, invasion, migration, and angiogenesis [6,7,8]. This knowledge led to the hypothesis on the anticancer properties of NSAIDs. Several studies, in vitro and in vivo, confirm the anticancer properties of NSAIDs. Although the role of COX2 inhibition in the anticancer properties of NSAIDs have been established, further studies reveal that the mechanism is more complex and that it cannot be explained by this hypothesis. It has been found that, in some cancer cells that are lacking COX2, NSAIDs had almost the same inhibitory effect on cancer cell growth and proliferation as in the other COX2-expressing cells [9,10,11,12]. Moreover, the concentration of NSAIDs required for cancer growth inhibition is much higher than those required for COX2 activity inhibition. Interestingly, studies on the chirality of NSAIDs show that the R-enantiomers of ibuprofen and flurbiprofen are lacking COX1 and COX2 inhibitory properties, compared to S-enantiomers, but that both of these enantiomers, simultaneously, had antiproliferative effects on cancer cells [13,14,15]. 

NSAIDs (i.e., ibuprofen, indomethacin) are a group of synthetic ligands of peroxisome proliferator-activated receptor-γ (PPARγ) [16]. PPAR belongs to the family of nuclear receptors with transcriptional activity. They exist in three isoforms: PPARα, PPARβ/δ, and PPARγ. In response to ligand activation, these receptors regulate adipogenesis, lipid metabolism, and glucose homeostasis. Furthermore, they are involved in inflammatory processes, proliferation, and cancer metabolism [17,18,19]. These receptors can be activated by natural ligands, such as fatty acids or prostaglandins, as well as by synthetic ligands [20,21,22,23]. The most popular and well-studied synthetic ligands of PPARγ are thiazolidinediones (i.e., troglitazone, pioglitazone), the class of drugs used for the treatment of type 2 diabetes mellitus [24,25,26]. Interestingly, they were found to provoke proapoptotic activities in cancer cells [25,27]. This finding has led to studies on the mechanism of PPARγ-induced apoptosis.

The activation of PPARγ leads to an increase in the expression of PRODH/POX [28]. Proline dehydrogenase/proline oxidase (PRODH/POX) (PRODH<, GenBank^TM^, NM_016335) is a flavin-dependent enzyme localized in the inner mitochondrial membrane [29,30]. This is the only cellular enzyme that degrades proline through its conversion into ∆^1^-pyrroline-5-carboxylate (P5C). This reaction generates free electrons, which are transported to the electron transport chain producing ATP, or they are accepted by oxygen-generating reactive oxygen species (ROS). In general, in energy-shortage conditions, PRODH/POX activity serves to supply energy by ATP production [29,31,32]. In some microenvironmental conditions, PRODH/POX is responsible for ROS-dependent apoptosis or autophagy [33,34]. PRODH/POX-dependent apoptosis undergoes cytochrome C release from the mitochondria to the cytosol, and the activation of caspase-9 and caspase-3 [34]. Therefore, PRODH/POX can play a dual role (prosurvival and proapoptotic); however, the mechanism for the switch between these processes is unknown. 

Proline conversion into P5C is coupled to the P5C reduction into proline, catalyzed by P5C reductase. This enzyme has three isoforms: PYCR1/PYCR2, which are specific for the mitochondrial compartment and the PYCRL present in cytosol. The conversion of P5C to proline, and its transport between mitochondria and cytosol, are also coupled with glucose metabolism by the pentose phosphate pathway [Rothwell, 2010, Long-term effect of aspirin on colorectal cancer incidence and mortality: 20-year follow-up of five randomised trials] [35,36]. Alternatively, P5C can be converted by P5C dehydrogenase (P5CDH) to glutamate, which is a precursor of α-ketoglutaric acid, which enters the TCA cycle [29]. This suggests that the proline-P5C cycle could play a key role in PRODH/POX-dependent apoptosis. It is known that PRODH/POX affects COX-2, MAPK, and EGFR, as well as the Wnt/β-catenin signaling pathways, linking these processes with the proapoptotic potential of PRODH/POX [37,38,39]. Another link leads to the p53 tumor suppressor protein, which is a potent PRODH/POX inducer [31,40,41]. The transcriptional regulation of PRODH/POX by p53 was found in the PRODH/POX promoter, containing a p53-response element [42]. 

In this report, we considered the NSAIDs to be agonists of PPARγ-induced PRODH/POX-dependent apoptosis in breast cancer MCF7 cells. Here, we present the results on an alternative COX2-independent mechanism of NSAID-induced apoptosis: the PRODH/POX stimulators in breast cancer MCF7 cells. 

## 2. Results

**Figure 1:**
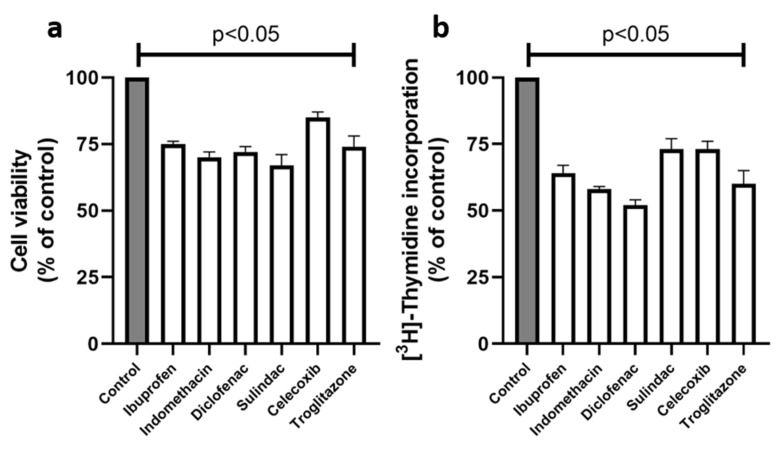
Cell viability (**a**) and DNA biosynthesis (**b**) in MCF7 cells treated for 24 h with the following concentrations of drugs: 0.75 mM ibuprofen; 0.5 mM indomethacin; 0.375 mM diclofenac; 0.06 mM sulindac; 0.02 mM celecoxib; and 0.02 mM troglitazone. The mean values ± standard error (SEM) from the 3 experiments, performed in duplicates, are presented at *p* < 0.05.

The studies on the NSAIDs, as ligands of PPARγ, were undertaken in order to establish the mechanism of their antineoplastic potential. Since NSAIDs are cytotoxic for cultured cells, a cell viability assay was performed. MCF7 breast cancer cells were treated for 24 h with NSAIDs, at the following concentrations: 0.750 mM of ibuprofen; 0.500 mM of indomethacin; 0.375 mM of diclofenac; 0.060 mM of sulindac; 0.020 mM of celecoxib; and 0.020 mM of troglitazone (as a positive control, standard agonist of PPARγ). As is shown in Figure 1a, the nonselective COX2 inhibitors, such as ibuprofen, indomethacin, diclofenac, and sulindac, decreased the MCF7 cell viability to 75, 70, 72, and 67% of the control, respectively. Celecoxib, as a selective COX2 inhibitor, decreased the cell viability to 85%, and troglitazone to 74% of the controls. The result of the experiment suggests that the drugs in the studied concentrations provoked low cytotoxicity in MCF7 cells. The concentrations of the studied drugs were used for further experiments.

Another parameter used for the evaluation of the cell viability under the effect of the drug treatment is DNA biosynthesis. As is shown in Figure 1b, NSAIDs significantly decreased the DNA biosynthesis in MCF7 cells. In the case of diclofenac, the process was decreased to 52%; in indomethacin, to 58%; in ibuprofen, to 64%; and in sulindac, to 73% of the control. Celecoxib and troglitazone inhibited DNA biosynthesis to 73% and 60% of the control, respectively. 

**Figure 2:**
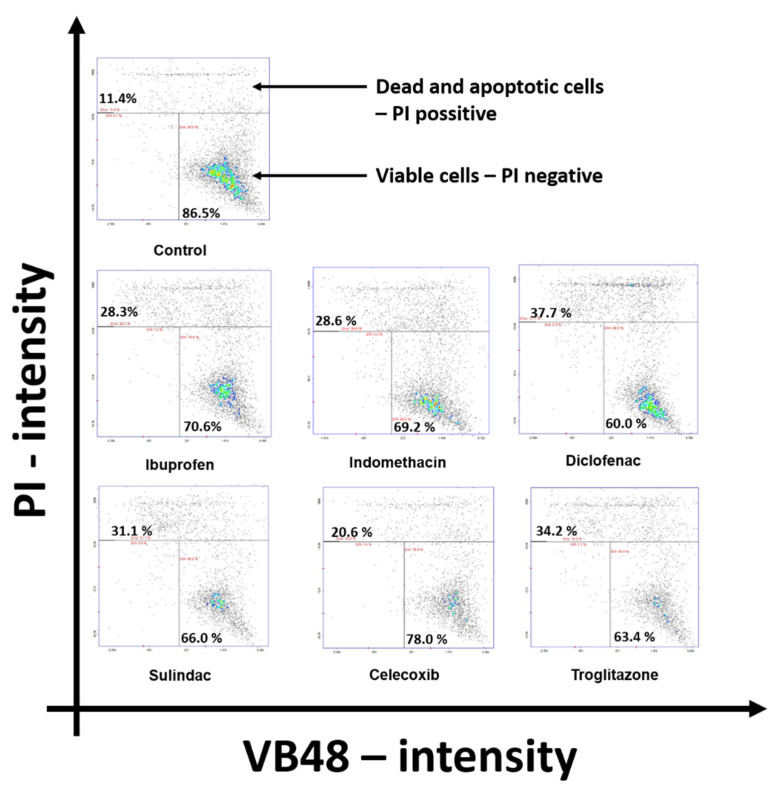
Cytometric assay of apoptosis in MCF7 cells. After 24 h of incubation with selected drugs (0.75 mM ibuprofen; 0.5 mM indomethacin; 0.375 mM diclofenac; 0.06 mM sulindac; 0.02 mM celecoxib; and 0.02 mM of troglitazone), the cells were stained with fluorescent dyes using Solution 5 (ChemoMetec^®^) containing Propidium Iodide and VB48. Cells in the upper polygon are apoptotic or dead. Cells in the lower right polygon are healthy living cells.

To evaluate the mechanism for the cytotoxicity of the studied drugs, the cytometric assay (ChemoMetec NucleoCounter), using Propidium Iodide and VB48 (Solution 5–ChemoMetec) dyes, was performed, as is shown in Figure 2. It has been found that the incubation of MCF7 cells with NSAIDs and troglitazone resulted in a decrease in the number of living cells, as compared to the control. The most significant increases in the numbers of dead cells were found in the cases of treatments with indomethacin, diclofenac, and troglitazone. The numbers of dead cells in these cases were: 28.3% for ibuprofen; 63.6% for indomethacin; 37.7% for diclofenac; 31.1% for sulindac; 20.6% for celecoxib; and 34.2% for troglitazone, respectively, versus 11.4% for the control. 

**Figure 3:**
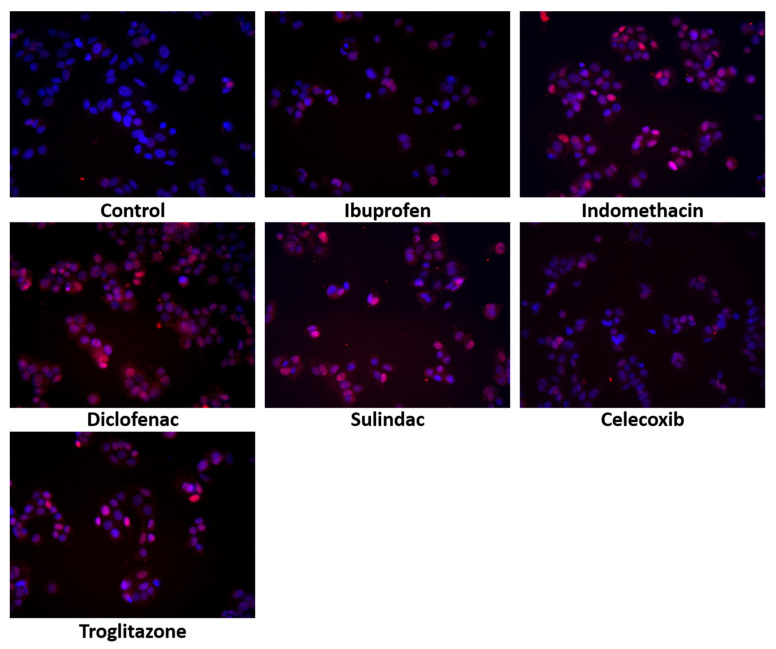
Immunofluorescence staning of p53 expression and its translocation to the nuceli in MCF7 cells treated for 24 h with the following drugs: 0.75 mM ibuprofen; 0.5 mM indomethacin; 0.375 mM diclofenac; 0.06 mM sulindac; 0.02 mM celecoxib; and 0.02 mM of troglitazone. Blue indicates the nuceli, and the red fluorescence represents p53 expression.

Although apoptosis is induced in response to various factors, the tumor suppressor protein, p53, is the most potent apoptosis activator. The expression of p53 and its translocation to the nucleus serve as a marker of apoptosis. As is shown in Figure 3, in the MCF7 cells treated with NSAIDs, the increase in the p53 expression was accompanied by their translocation to the nuclei. This was particularly pronounced in the cells treated with indomethacin, diclofenac, sulindac, and troglitazone.

**Figure 4:**
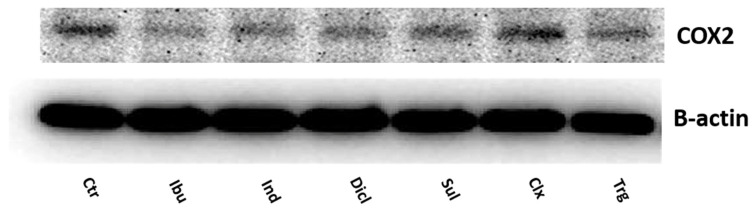
Western blot analysis of COX2 expression in MCF7 cells after 24 h of incubation, with 0.75 mM ibuprofen; 0.5 mM indomethacin; 0.375 mM diclofenac; 0.06 mM sulindac; 0.02 mM celecoxib; and 0.02 mM of troglitazone. For the experiment, 40 µg/lane of protein from the lysates was used. The expression of Β-actin serves as a loading control.

Although the proapoptotic activity of NSAIDs could undergo COX2 inhibition, in the case of celecoxib, a selective COX2 inhibitor, it is unlikely since it was ineffective in the inhibition of the expression of COX2 in the MCF7 cells in Figure 4. 

**Figure 5:**
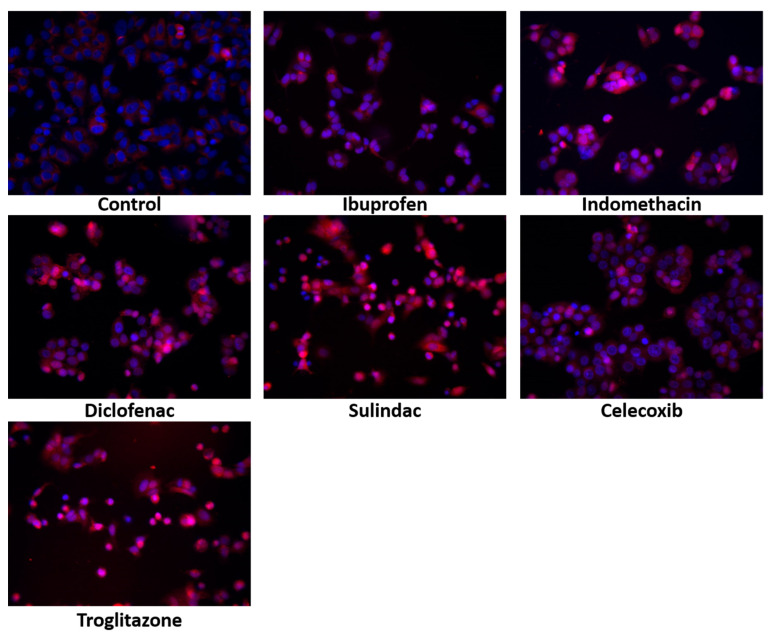
Immunofluorescence staining of PPARγ and its translocation to the nuclei in MCF7 cells treated for 24 h with the following drugs: 0.75 mM ibuprofen; 0.5 mM indomethacin; 0.375 mM diclofenac; 0.06 mM sulindac; 0.02 mM celecoxib, and 0.02 mM troglitazone. Blue indicates the nuceli, and the red fluorescence represents PPARγ localization and expression.

**Figure 6:**
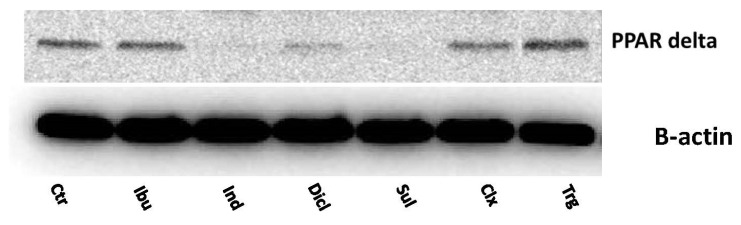
Western blot analysis for PPARδ expression in MCF7 cells incubated for 24 h with the following drugs: 0.75 mM ibuprofen; 0.5 mM indomethacin; 0.375 mM diclofenac; 0.06 mM sulindac; 0.02 mM celecoxib; and 0.02 mM troglitazone. For the experiment, 40 µg/lane of protein from the lysates was used.

The proapoptotic potential of troglitazone led to the study of the impact of NSAIDs on PPARγ expression. It is well known that NSAIDs are ligands of the PPARγ. In MCF7 cells, the NSAIDs were found to upregulate the expression of PPARγ and its translocation to the nucleus (Figure 5). Simultaneously, indomethacin, diclofenac, and sulindac decreased the expression of PPARδ, as compared to the control (Figure 6). This suggests that the proapoptotic activity of NSAIDs is mediated by PPARγ.

**Figure 7:**
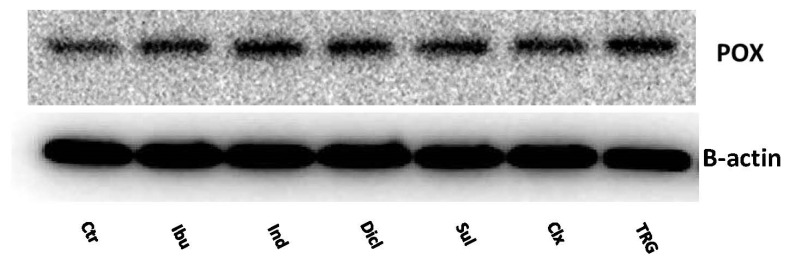
Western blot for PRODH/POX expression in MCF7 cells treated for 24 h with the following drugs: 0.75 mM ibuprofen; 0.5 mM indomethacin; 0.375 mM diclofenac; 0.06 mM sulindac; 0.02 mM celecoxib; and 0.02 mM troglitazone. For the experiment, 40 µg/lane of protein lysates was used.

One of the effects of PPARγ activation is the increased expression of PRODH/POX. PPARγ ligands (i.e., thiazolidinediones) are known to induce apoptosis via the mitochondrial activation pathway. The Western Blot analysis for PRODH/POX shows that, in the MCF7 cells treated with all of the studied drugs, the expression of PRODH/POX was increased, compared to the control in Figure 7. 

**Figure 8:**
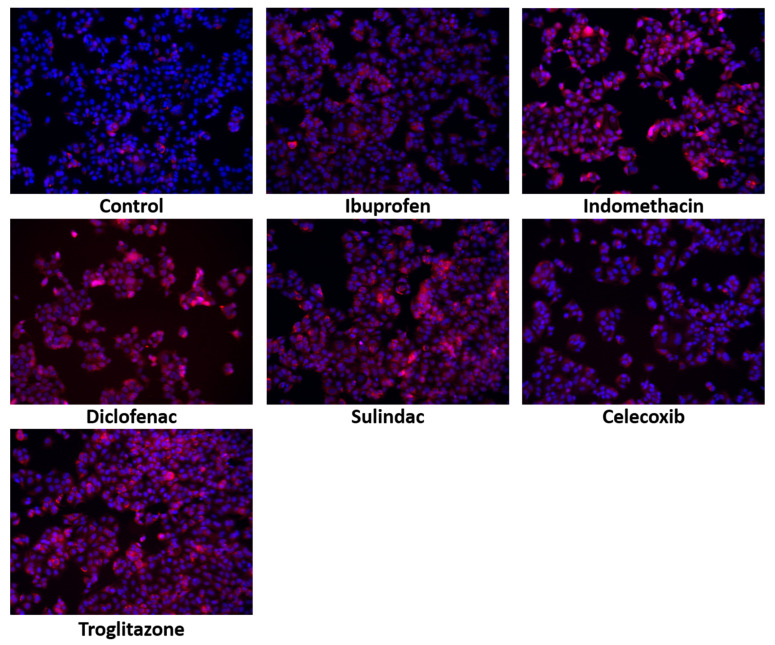
Immunofluorescence staining of PRODH/POX expression in MCF7 cells treated for 24 h with the following drugs: 0.75 mM ibuprofen; 0.5 mM indomethacin; 0.375 mM diclofenac; 0.06 mM sulindac; 0.02 mM celecoxib; and 0.02 mM troglitazone. Blue stainng indicates the nuceli, and red staining represents PRODH/POX expression.The inhibition of DNA biosynthesis by the studied drugs was accompanied by the inhibition of protein biosynthesis. The biosynthesis of collagen, the most abundant protein in mammalian cells, can represent the total protein synthesis. As is presented in Figure 9a, NSAIDs and troglitazone provoke strong inhibitory effects on collagen biosynthesis in MCF7 cells. In the case of nonselective COX2 inhibitors, such as ibuprofen, indomethacin, diclofenac, and sulindac, the process was inhibited to 32, 28, 21, and 25% of the control, respectively. The selective COX2 inhibitor, celecoxib, and the PPARγ agonist, troglitazone, decreased the collagen biosynthesis to 27% and 19% of the control, respectively.

Additionally, the immunofluorescence of PRODH/POX confirmed its upregulation in response to the studied drugs, as is shown in Figure 8.

**Figure 9:**
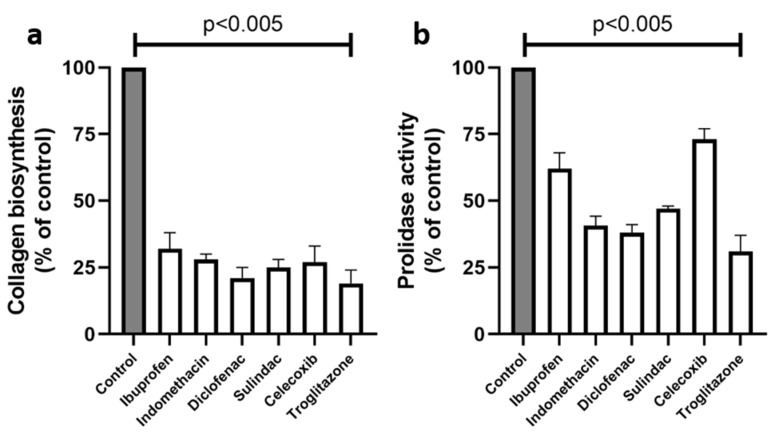
Collagen biosynthesis (**a**) and prolidase activity (**b**) in MCF7 cells treated for 24 h with the following concentrations of drugs: 750 µM ibuprofen; 500 µM indomethacin; 375 µM diclofenac; 60 µM sulindac; 20 µM celecoxib, and 20 µM troglitazone. The mean values ± standard error (SEM) from the 3 experiments, performed in duplicates, are presented at *p* < 0.005.

Collagen biosynthesis is coupled to the activity of prolidase, the enzyme supporting proline for the process. As is shown in Figure 9b, NSAIDs significantly inhibited the activity of prolidase. The incubation of MCF7 cells, for 24 h, with ibuprofen, indomethacin, diclofenac, and sulindac, resulted in prolidase activity inhibition to 62, 41, 38, and 47% of the control, respectively. Troglitazone provoked a strong inhibitory potency on the prolidase activity (31% of control), while celecoxib inhibited the enzyme to 73% of the control. 

**Figure 10:**
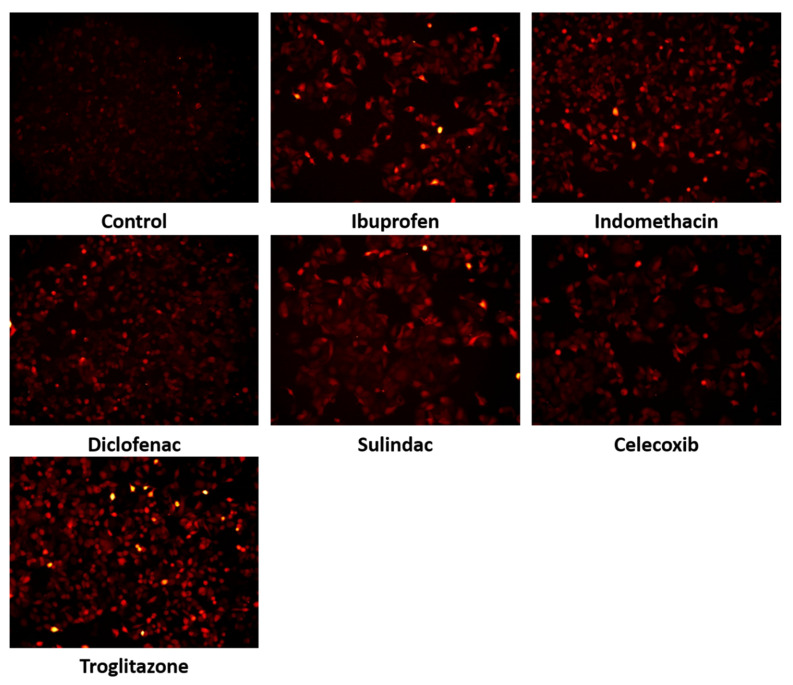
Fluorescence analysis of reactive oxygen species generation due to the POX activation using 0.5 µM of 2′,7′-dichlorofluorescin diacetate staining. Analysis performed on live cells using a survival chamber: 37 °C in 5% CO_2_. MCF7 cells were incubated with the following drugs: 0.75 mM ibuprofen; 0.5 mM indomethacin; 0.375 mM diclofenac; 0.06 mM sulindac; 0.02 mM celecoxib; and 0.02 mM troglitazone, for the 4 h. Red fluorescence intensity represents amount of generated ROS.

Since PPRODH/POX-dependent apoptosis is associated with ROS generation, the MCF7 cells were treated with NSAIDs and troglitazone and were incubated with DCFDA. The increased level of fluorescence in the cells is due to the oxidation of DCFDA to its fluorescence form, and it can be considered an indicator of ROS generation. As is shown in Figure 10, in MCF7 cells treated with NSAIDs and troglitazone, a significant increase in ROS generation was found. This suggests that the oxidative stress induced by all the studied drugs led to apoptosis activation and cell death. 

**Figure 11:**
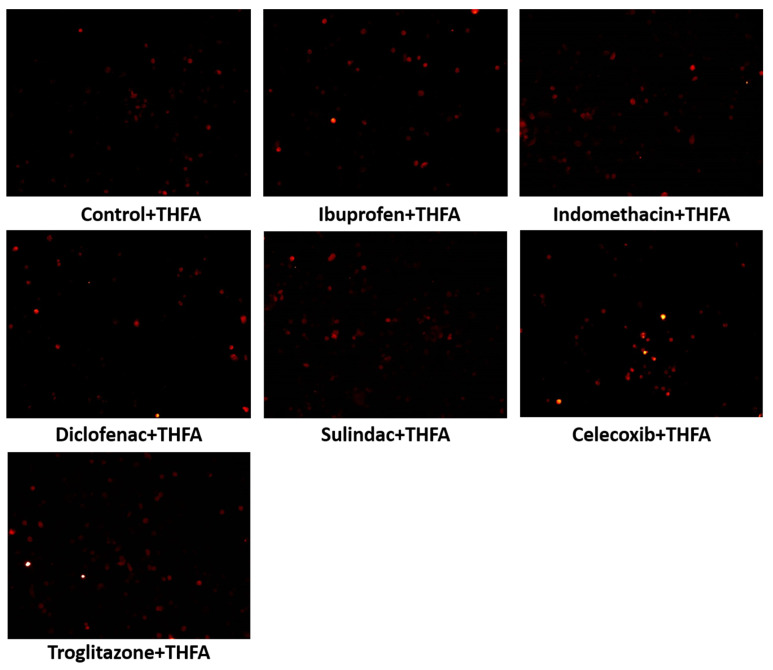
Fluorescence analysis of ROS generation in MCF7 in the presence of THFA (PRODH/POX inhibitor). NSAID POX activation was inhibited by 0.5 h preincubation, with 0.1 mM of THFA. Fluorescence was determined by using 0.5 µM of 2′,7′-dichlorofluorescin diacetate staining. Analysis was performed on live cells using a survival chamber: 37 °C in 5% CO_2_. After preincubation with THFA, the MCF7 cells were incubated with the following drugs: 0.75 mM ibuprofen; 0.5mM indomethacin; 0.375 mM diclofenac; 0.06 mM sulindac; 0.02 mM celecoxib; and 0.02 mM troglitazone, for the 4 h. Red fluorescence intensity represents amount of generated ROS.

In the MCF7 cells, in which PRODH/POX was inhibited by THFA, ibuprofen, indomethacin, diclofenac, sulindac, celecoxib, and troglitazone, ROS generation did not increase, as is shown in Figure 11.

**Figure 12:**
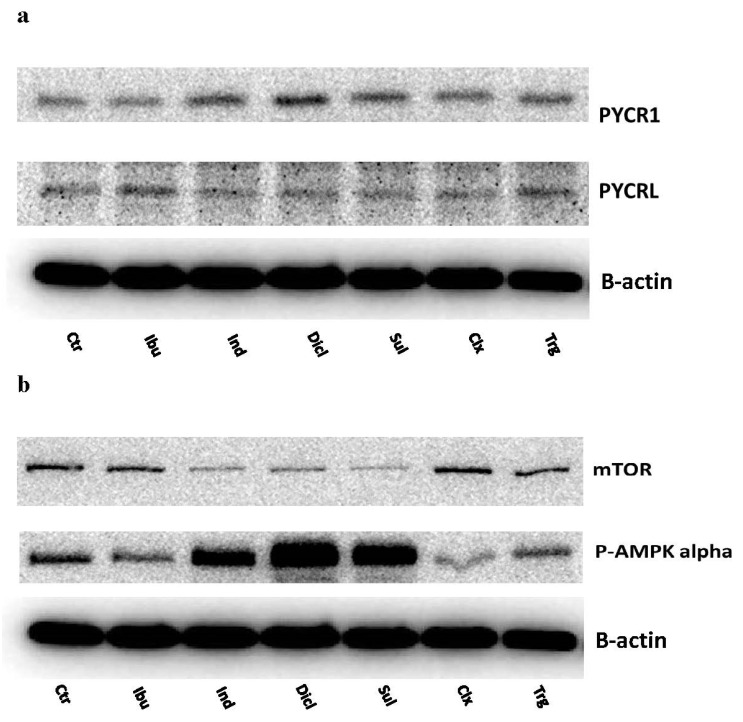
Western blot for PYCR1 and PYCRL (**a**), and mTOR and p-AMPK alpha (**b**) expressions in MCF7 cells treated for 24 h with the following drugs: 0.75 mM ibuprofen; 0.5 mM indomethacin; 0.375 mM diclofenac; 0.06 mM sulindac; 0.02 mM celecoxib; and 0.02 mM troglitazone. For the experiment, 40 µg/lane of protein lysates was used.

Interestingly, indomethacin, diclofenac, and sulindac, which enhanced the PRODH/POX expression, were also responsible for the increase in the expression of PYCR1, a mitochondrial enzyme that is responsible for the conversion of P5C to proline. At the same time, the cytosolic form of this enzyme, called PYCRL, did not show any significant changes in the expression in response to NSAIDs and troglitazone (Figure 12a). These data support the hypothesis that NSAIDs induce ROS generation by the rapid turnover of proline in the mitochondria. 

One of the master regulators of cell metabolism is mTOR and AMPK. In MCF7 cells, indomethacin, diclofenac, and sulindac inhibited mTOR expression, while troglitazone provoked a weaker effect, as compared to the control. Interestingly, the effect was coupled to the NSAID-induced expression of p-AMPK alpha. This suggests the role of energetic processes in the mechanism of NSAID-induced cell death (Figure 12b). 

**Figure 13:**
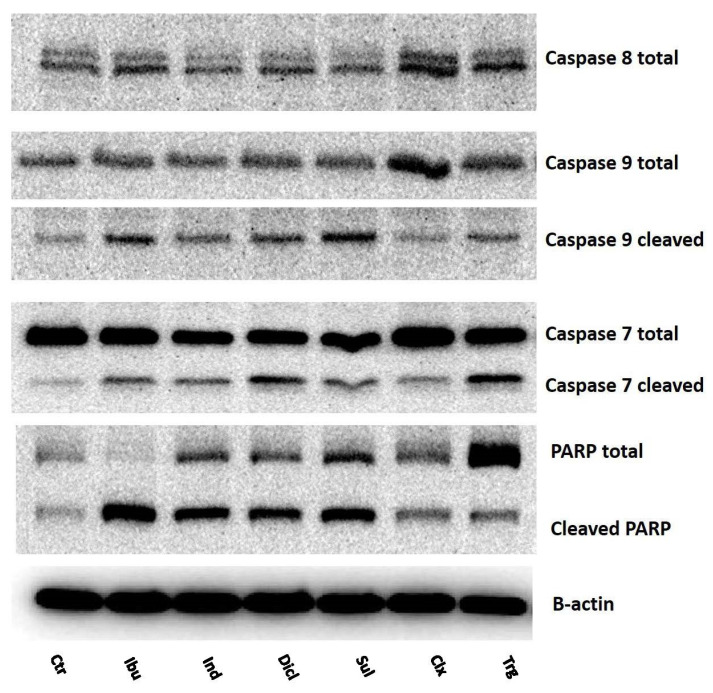
Western blot for caspase-8, caspase-9, and caspase-7 (total and cleaved), PARP, total and cleaved, in MCF7 cells treated for 24 h with the following drugs: 0.75 mM ibuprofen; 0.5 mM indomethacin; 0.375 mM diclofenac; 0.06 mM sulindac; 0.02 mM celecoxib; and 0.02 mM troglitazone. For the experiment, 40 µg/lane of protein lysates was used. Protein expression was normalized versus β-actin expression.

To verify the hypothesis, that NSAIDs induce PRODH/POX-dependent apoptosis, the expressions of some of the markers of apoptosis were evaluated in the NSAID-treated MCF7 cells. A representative group of apoptosis markers are caspases, the enzymes present in normal cells in a nonactive (proenzymatic) state. In response to the apoptosis signals, i.e., DNA damage and oxidative stress, proenzymes are cleaved to the active form, initiating a cascade of signaling pathways, which are terminated by cell death. Caspase-8 is involved in the extrinsic apoptosis pathway, which is associated with the activation of death receptors on the cell membrane in response to different ligands. In the MCF7 cells treated with NSAIDs, only celecoxib induced the expression of caspase-8, compared to the control (Figure 13). However, the cleaved form of caspase-8 was not detected in any of the investigated samples. The expression of caspase-9 is induced when apoptosis is activated by the intrinsic (mitochondrial) pathway. We have found that ibuprofen, indomethacin, diclofenac, sulindac, and troglitazone increased the expression of cleaved caspase-9, showing that NSAID-induced apoptosis is due to the mitochondrial pathway. In the case of celecoxib, in spite of the increased expression of caspase-9 (proenzyme), there were no significant changes in the cleaved form of caspase-9, compared to the control. Caspase-7 is an effector caspase that is activated in response to extrinsic or intrinsic apoptotic signals. Our experiment shows that caspase-7 is expressed in MCF7 cells. In the cells treated with NSAIDs, the expression of cleaved caspase-7 was increased, compared to the control. However, celecoxib had no influence on the cleaved caspase-7 expression. Another apoptosis marker is PARP, poly (ADP-ribose) polymerase, which is important for DNA repair and is activated by limited cleavage during environmental-stress-induced DNA damage. We have found that NSAIDs, except for celecoxib and troglitazone, increased the expression of cleaved PARP, supporting the proapoptotic activity of NSAIDs. 

## 3. Discussion

Although the anticancer properties of NSAIDs has been observed in different in vitro/in vivo models, as well as in clinical practice, the mechanism for the activity is not well recognized [43,44]. Some studies suggest that the underlying mechanism involves COX2 inhibition. In fact, COX2 is overexpressed in most cancer cells [5]. The postulated anti-inflammatory mechanism of the proapoptotic properties of NSAIDs seems to be not the only one, since the cells lacking COX2 also responded to NSAID-induced apoptosis, suggesting a COX2-independent mechanism [9,10,12,13,14,45,46,47,48].

In the present study, we hypothesize that NSAIDs could induce apoptosis as PPARγ agonists that activate PRODH/POX. This hypothesis has strong experimental foundations, since NSAIDs, including celecoxib, are ligands of PPARγ [49], and PPARγ has a documented strong potency for the activation of PRODH/POX [28]. The rationale for studies on celecoxib is that this drug is a selective COX2 inhibitor, and it fits perfectly into the hypothesis of the COX2-independent antineoplastic activities of NSAIDs. Furthermore, this drug has been proved as a PPARγ agonist. Therefore, we used this drug as a positive control for COX2-dependent apoptosis. The other NSAIDs used in our research, namely, ibuprofen, indomethacin, diclofenac, and sulindac, are nonselective COX2 inhibitors, and have been proven to evoke anticancer activities with regard to COX2 inhibition. Moreover, this nonselective COX2 inhibitor has well-documented agonistic activity against PPARγ, which makes it an attractive tool in the evaluation of NSAID-induced apoptosis via the PPARγ–PRODH/POX axis.

PRODH/POX participate in the generation of ATP or ROS, depending on the environmental conditions. Since cancer cells have high energy requirements, and glucose does not produce enough energy because of Warburg’s effect, the cells use proteins (mainly collagen) as an additional source of energy. The exceptional product of this degradation is proline. The proline is degraded in the mitochondria by the yielding of pyrroline-5 carboxylate (P5C) by PRODH/POX. During this process, ROS are generated, inducing apoptosis [29,33,34,37,50]

Such a scenario likely occurs in the case of breast cancer cells treated with NSAIDs because, in such conditions, increases in the expressions of AMPK and mTOR were observed, especially in the cases of the treatments of the cells with indomethacin, diclofenac, and sulindac. Moreover, we observed the inhibition of anabolic processes in the NSAID-treated MCF7 cells. All of the studied drugs inhibited collagen biosynthesis, the process that utilizes proline. Therefore, in such conditions, proline is available for the PRODH/POX-dependent function.

Our hypothesis, that PRODH/POX-dependent apoptosis is induced by PPARγ activated by NSAIDs, is highly possible since we found that these drugs activated both the extrinsic and intrinsic pathways of apoptosis. However, in the cases of ibuprofen, indomethacin, diclofenac, and sulindac, apoptosis was due to the mitochondrial pathway (activation of caspase-9) and was strongly correlated with PRODH/POX expression and ROS generation.

The proapoptotic potential of PRODH/POX is supported by evidence that the gene for PRODH/POX is p53 inducible [39]. In fact, the results of our study show that, in MCF7 cells treated with various NSAIDs, the p53 expression is drastically increased with their translocation to the nuclei. Moreover, the hypothesis that NSAID-induced PRODH/POX-dependent apoptosis undergoes PPARγ is supported by the experiment showing the increased expression and translocation of the PPARγ receptors to the nuclei. 

Of some importance could also be the study on the role of PEPD in the sequestration and inactivation of the transcriptional activity of p53 [51]. In our study, we found that the NSAID-treated cells showed an increase in the p53 expression and a decrease in the PEPD expression. Such conditions, therefore, favor the proapoptotic function of p53. Even in the case of the formation of the p53-PEPD complex, during stress conditions such as oxidative stress, p53 can be released from the complex, recovering the transcriptional activity [51]. 

Another intriguing question relates to the mechanism for PRODH/POX-dependent ROS formation. It could be suggested that the NSAID-induced increase in the expression of PRODH/POX accelerates the proline degradation into P5C, which could be reconverted to the proline by the P5C-reductase (PYCR). This processes is well known as a “proline cycle” [35]. Two isoenzymes of PYCR are known: mitochondrial PYCR1 and cytoplasmic PYCRL. We found that, in NSAID-treated MCF7 cells, cytosolic PYCRL was not affected, while the mitochondrial variant of this enzyme (PYCR1) was upregulated. It seems that the upregulations of PRODH/POX and PYCR1 accelerates the mitochondrial proline turnover, releasing ROS-induced apoptosis. Whether this is the case requires further study. 

The application value of the results presented in this paper have some limitations that are due to the in vitro nature of the studies and the high doses of NSAIDs. Although the cell line models have some limitations (e.g., the inability to observe systemic phenomena), they are a powerful tool, which offer several advantages. Certainly, the cell models allow for the strict control of the conditions of the experiment in order to establish the critical factor affecting the studied processes in a relatively short time. They are especially helpful in cases of a limited availability of clinical samples or in vivo models. Therefore, the results on the cell models allow for the prediction of the consequences of the pharmacotherapeutic manipulation in humans, and they provide a rationale for clinical studies on the dose-dependent effects. Different treatment regimens and combinations of therapies have been evaluated using cell lines, which have yielded interesting, and potentially promising, results. It seems that some of them could have an application value. However, NSAID treatment may contribute to several toxic effects, particularly at high doses. Because of the NSAID-dependent inhibition of cyclooxygenases (particularly COX1), various effects may occur, such as: the exacerbation of hypertension, fluid retention, gastrointestinal complications, and cardiovascular events. Prostaglandins, prostacyclins, and thromboxane, produced in response to COX1 activity, are the most potent homeostasis regulators for the physiological functions of the gastrointestinal tract, the blood clotting process, and the cardiovascular system, generally influencing the physiological balance in the human body. As a result of the improper or overdose intake of some NSAIDs, toxic effects can occur. Some of the most common include: coagulopathy, upper gastrointestinal bleeding or perforation, dyspepsia, mucosal damage, hepatotoxicity, fatal liver injury, myocardial infarction, heart failure, and hypertension [52]. This information seems controversial considering the fact that most of these drugs are classified as OTC and are commonly used for pain and inflammation treatment therapies. Nevertheless, in vivo studies are required to establish the effective doses of NSAIDs for cancer treatment, minimizing the side effects of their actions

**Figure 14:**
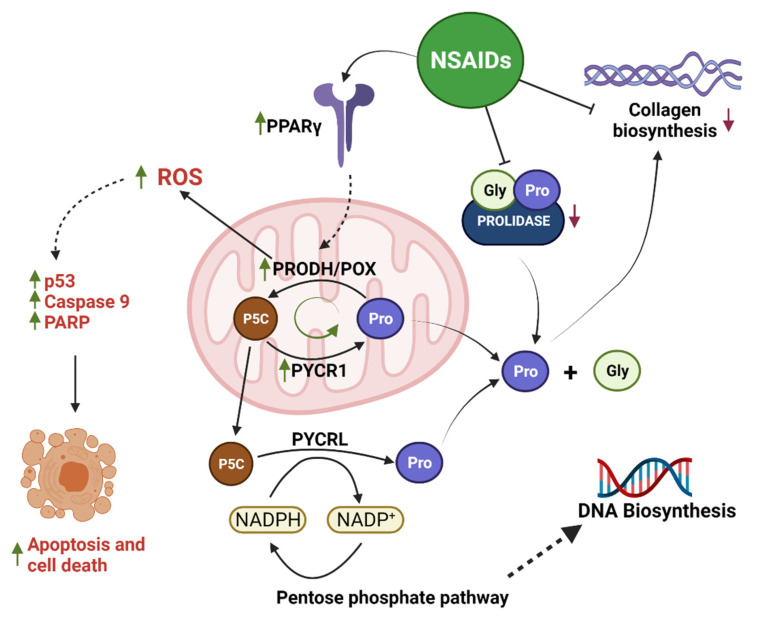
The mechanism of PRODH/POX-dependent apoptosis induced by NSAIDs as PPARγ agonists. Created with BioRender.com, access on 29 December 2021.

The data presented in this report provide an explanation for the mechanism of NSAID-induced apoptosis in breast cancer MCF7 cells. We suggest that NSAIDs activate PPARγ, which upregulates PRODH/POX-induced ROS formation and apoptosis. The potency of NSAIDs in this regard is facilitated by their ability to inhibit collagen biosynthesis, the main utilizer of proline. In this way, proline is supported as a substrate for PRODH/POX, accelerating proline cycling and ROS formation. This suggests that some of the NSAIDs that provoke strong collagen-biosynthesis-inhibiting activity could be considered effective anticancer agents. The graphical visualization of mechanism for NSAID-s induced apoptosis is presented as Figure 14.

## 4. Materials and Methods 

### 4.1. Materials

MCF7 (ATCC^®^ HTB-22™) were obtained from the ATCC. Horseradish-peroxidase-conjugated anti-rabbit IgG and anti-mouse IgG antibodies, Alexa488 conjugated anti-rabbit IgG, bacterial collagenase, 3-(4,5-dimethylthiazole-2-yl)-2,5-diphenyltetrazolium bromide (MTT), DCFDA, tetrahydro-2-furoic acid (THFA), glycyl-L-proline, ibuprofen, indomethacin, diclofenac, and sulindac were purchased from Sigma-Aldrich. The celecoxib and troglitazone were products of Targetmol. Solution 5 (VB-48™ PI–AO) was purchased from ChemoMetec. The primary antibodies against: COX2; p53; p-AMPKα; m-TOR; caspase-8; caspase-9, total and cleaved; caspase-7, total and cleaved; PARP, total and cleaved; and B-actin were products of Cell Signaling Technology. The PRODH/POX primary antibodies and PPARγ were products of Santa Cruz Biotechnology. The PYCR1, PYCRL, and PPAR delta primary antibodies were obtained from Abnova. The Hoechst 33342 was obtained from Becton Dickinson. 

### 4.2. Methods

#### 4.2.1. Cell Culture 

MCF7 (ATCC^®^ HTB22™) breast cancer cells were maintained in the MEM Eagle’s medium, supplemented with 10% FBS, 50 U/mL of penicillin, and 50 µg/mL of streptomycin, and incubated at 37 °C in 5% CO_2_. The cells were grown on 100-mm dishes in 10 mL of complete medium. The cell culture medium was changed 2–3 times per week. For the experiments with the investigated drugs, we used MEM Eagle’s medium without FBS and Pen/Strep.

#### 4.2.2. Cell Viability Test MTT

To evaluate the cytotoxicity of the selected drugs on the MCF7 cells, methyl thiazolyl tetrazolium (MTT) salt was used, as described in the Carmichael method [53]. This method is based on the conversion of yellow tetrazolium bromide MTT solution to the purple formazan derivatives in the live cells, and this is due to the activity of the mitochondrial dehydrogenases. For this assay, the cells were cultured in 12-well plates, at a density of 0.1 × 10^6^ cells/well. When the cells reached about 70% of confluency, the culture media were removed. The wells were washed with PBS, and the fresh MEM Eagle’s media containing the studied drugs, dissolved in DMSO (dimethyl sulfoxide), were added into the wells (concentration of DMSO in sample did not exceed 0.1%). The samples were prepared in triplicates. The cells were incubated with the compound for 24 h. The investigated drugs were in the following concentrations: 0.750 mM of ibuprofen; 0.500 mM of indomethacin; 0.375 mM of diclofenac; 0.060 mM of sulindac; and 0.020 mM of celecoxib. An amount of 0.020 mM of troglitazone was used as a positive control (PPARγ agonist). After incubation, the cell culture media containing the studied drugs was removed, and the plates were washed twice with prewarmed PBS. Furthermore, the cells were incubated at 37 °C for 1 h, with MTT dissolved in PBS (0.5 mg/mL) in a volume of 1.0 mL per well. When the incubation step ended, the MTT was removed and the formazane derivatives were dissolved in the DMSO (1.0 mL per well). To quantify the formed formazane, the absorbance at 570 nm was measured using a spectrophotometer. The viability of the cells treated with the studied drugs was calculated as a percent of the control value.

#### 4.2.3. DNA Biosynthesis 

To study the effect of NSAIDs and troglitazone on the DNA biosynthesis, an assay of the radioactive [^3^H]-Thymidine incorporation into the DNA was performed. The cells were cultured on 12-well plates, as described above. When they reached about 70% of confluency, they were treated with the studied drugs in the described concentrations, and 1.0 μCi of [^3^H]-Thymidine was added to the MEM Eagle’s medium. The samples were prepared in triplicates. The cells were incubated for 24 h at 37 °C in 5% CO_2_. The radioactivity of the samples was measured using the Tri-Carb 2810 TR Scintillation Analyzer (PerkinElmer, Waltham, MA, USA). To calculate the DNA biosynthesis, the DPMI values of the treated cells were compared to those of the control samples. 

#### 4.2.4. Collagen Biosynthesis

The collagen biosynthesis was evaluated by measuring the incorporation of the radioactive 5-[^3^H]-Proline into the collagen. The MCF7 cells were cultured in 12-well plates. When the cells were about 70% confluent, the culture medium was removed, and the cells were treated for 24 h with the studied drugs and 5- [^3^H]-Proline (5 μCi/mL). Digesting proteins, with purified *Clostridium histolyticum* collagenase, were used to determine the incorporation of the tracer into the collagen, as was described by Peterkofsky et al. [54]. The radioactivity of the samples was measured by the Tri-Carb 2810 TR Scintillation Analyzer (PerkinElmer, Waltham, MA, USA). To calculate the collagen biosynthesis, the DPMI values of the treated cells were compared to those of the controls. 

#### 4.2.5. SDS PAGE and Western Blotting

For the analysis of the protein expression by Western blotting, the cells were cultured in 100-mm plates, at about 2.0 × 10^6^ cells, and when they reached about 70–80% of confluency, the culture medium was removed, and the cells were treated with the studied drugs (dissolved in culture medium). After 24 h of incubation, the culture media were removed, and the cells were harvested using a cell lysis buffer supplemented with a protease/phosphatase inhibitor cocktail. The protein concentrations of the samples were determined by the Lowry method [55]. Then, the proteins were separated using the SDS-PAGE method, described by Laemmli [56]. After this step, the gels were washed in the cold Towbin buffer (25 mM Tris, 192 mM glycine, 20% (*v*/*v*) methanol, 0.025–0.1% SDS, pH 8.3). The proteins in the gels were transferred onto the 0.2-µm nitrocellulose membranes by Trans-Blot (BioRad, Hercules, CA, USA). The transfer conditions were 100 mA, 1 h in freshly prepared Towbin buffer, and the temperature was maintained around 4–8 °C. The blocking of the membranes was performed by 5% NFDM, for 1 h, at RT. When the blocking was complete, the membranes were washed three times with 20 mL of TBS-T (20 mM Tris, 150 mM NaCl, and 0.1% Tween^®^ 20). After the washing step, the membranes were incubated with primary antibodies overnight at 4 °C. The concentration of the primary antibodies was 1:1000. Furthermore, the membranes were washed three times with 20 mL of TBS-T, and a secondary antibody, conjugated with HRP solutions (1:3000) in 5% NFDM, was used for 1 h, at RT. Then, the membranes were washed 3 times, with 20 mL of TBS-T, and visualized. 

#### 4.2.6. ROS Formation

The cells were cultured on black wells in a 96-well plate, at 0.01 × 10^4^ cells/well. When the cells reached 70–80% confluency, the culture media were removed, the plate was washed with PBS, and 100 µL of medium containing the studied drug was added into the well. After 4 h of incubation, 0.5 µM of 2′,7′-dichlorofluorescin diacetate was added to the wells and was incubated for 15 min, at 37 °C, in 5% CO_2_. After incubation, the culture media with DCFDA were removed and the cells were washed twice with prewarmed PBS. Then, the wells were loaded with 100 µL of PBS. The cells were visualized with the BD Pathway 855 Bioimaging system, in an environmental control chamber (37 °C in 5% CO_2_), and ex. λ 488 nm, and em. λ 521 nm. In its basic state, DCFDA is a nonfluorescent compound, and, when oxidized by ROS to DCF, it becomes highly fluorescent. 

#### 4.2.7. Cytometric Assay for Apoptosis 

The cells were cultured in 6-well plates at an initial density of 0.6 × 10^6^ cells/well. When the cells reached about 70–80% of confluency, the culture media were removed. The wells were washed with PBS, and fresh MEM Eagle’s media containing the studied drugs were added into the wells. After 24 h of incubation, the cells (including floating cells) were collected by trypsinization to 1.5-mL Eppendorf tubes. The cells were centrifuged at 1000× *g* for 5 min. The supernatant was removed and the cell pellet was resuspended in 190 µL of PBS, and 10 µL of Solution 5 (Acridine Orange, Propidium Iodide and VB-48) was added. The cell suspension with staining dyes, of 10 µL, was loaded onto the chambers on the NC-Slide A8, and the slide was read by the Vitality Protocol. Cells with low fluorescence intensities of PI (PI negative), and high fluorescence intensities of VB48, represent living cells with high viability. The cells with high fluorescence intensities of PI (PI positive) represent dead cells. 

#### 4.2.8. Immunofluorescent Analysis

The cells were cultured on black wells on a 96-well plate, at 0.01 × 10^4^ cells/well. After 24 h, the culture media were removed, the plate was washed with PBS, and 100 µL of the medium containing the studied drug was added into the well. After 24 h, the culture media containing the drugs were removed, and the cells were fixed with a 3.7% formaldehyde solution at room temperature for 10 min. Then, the plate was washed once with 100 µL/well of PBS. Later, the permeabilization, with a 0.1% Triton X-100 solution–10 min step, was performed. After the permeabilization, the plate was washed twice with PBS, and 3% FBS was used as a blocking agent at room temperature for 30 min. After the removal of 3% FBS, 50 µL of the primary antibody (1:50), diluted in 3% FBS, was added, and the plate was incubated for one hour at room temperature. After incubation, the primary antibody plate was washed three times with PBS. Then, 50 µL per well of the secondary antibody (dilution 1:1000) was added for the 1 h. During this step, the plate was covered from the light. When the secondary antibody solution was removed, the plate was washed 3 times with PBS, and the wells were filled with 100 µL of PBS containing 2 µg/mL of Hoechst 33,342 for the nuclei staining. The plate was visualized using the BD Pathway 855 Bioimaging system.

#### 4.2.9. Prolidase Activity 

For this experiment, the cells were cultured in the 100-mm plates (about 1 × 10^6^ cells), and when they reached about 70–80% of confluency, the culture medium was removed, the cells were rinsed with PBS, and the culture medium, *w*/*o* FBS and Pen/Strep, was added. The cells were treated for 24 h with the studied drugs. After the incubation, the cells were collected and subjected to a prolidase assay, according to the method of Myara [57]. For the determination of the total protein concentration in the samples, the method of Lowry was used [55]. The prolidase activity was reported as the nanomoles of proline released from the synthetic substrate (Gly-Pro), for one minute, per milligram of the supernatant protein of the cell homogenate. 

#### 4.2.10. Statistical Analysis

In order to analyze the distribution of the data in individual groups, the Shapiro–Wilk test was performed. Within the normally distributed groups, the differences between the individual groups were analyzed using a one-way ANOVA, with multiple post hoc comparisons using the Bonferroni correction. In the groups where no normal distributions were shown, the nonparametric Mann–Whitney test was used. The data are presented as means ± standard error of measurement (SEM).

## 5. Conclusions

Our report provides new insights into the mechanism of the anticancer activity of NSAIDs. We have found that NSAIDs, as PPARγ agonists, activate PRODH/POX-induced ROS-dependent apoptosis in breast cancer MCF7 cells. The mechanism involves the NSAID-dependent inhibition of collagen biosynthesis (the main proline-consuming process), providing a substrate for PRODH/POX-dependent functions. This suggests that targeting proline metabolism and the PRODH/POX–PPARγ axis can be considered a novel approach for breast cancer treatment.

## Data Availability

This study did not report any supporting data.

## Abbreviations

AMPK	AMP-activated protein kinase
ATP	adenosine triphosphate
COX	cyclooxygenase
EGFR	epidermal growth factor receptor
MAPK	mitogen-activated protein kinase
mTOR	mechanistic target of rapamycin
NSAIDs	nonsteroidal anti-inflammatory drugs
P5C–∆^1^	pyrroline-5-carboxylate
P5CDH	P5C dehydrogenase
PARP	poly (ADP-ribose) polymerase
PGE_2_	prostaglandin E_2_
PPAR	peroxisome proliferator-activated receptor
PRODH/POX	proline dehydrogenase/proline oxidase
PYCR1	mitochondrial P5C reductase
PYCRL	cytosolic P5C reductase
ROS	reactive oxygen species
